# Crosstalk between arachidonic acid metabolism and glycolysis drives integrated metabolic-inflammatory reprogramming in macrophages

**DOI:** 10.7150/ijbs.116671

**Published:** 2026-01-01

**Authors:** Yuanyuan Cheng, Ni Fan, Xiuying Zhang, Wei Zhao, Baoping Xie, Jia Zhao, Marlene Rong, Xuechen Li, Hung-Fat Tse, Jianhui Rong

**Affiliations:** 1 Guangdong Key Laboratory for Translational Cancer Research of Chinese Medicine, Key Laboratory of Chronic disease Prevention and Control of Traditional Chinese Medicine of Guangdong Higher Education Institutes, International Institute for Translational Chinese Medicine, School of Pharmaceutical Sciences, Guangzhou University of Chinese Medicine, Guangzhou, Guangdong, 510006, China.; 2 School of Chinese Medicine, Li Ka Shing Faculty of Medicine, The University of Hong Kong, 10 Sassoon Road, Pokfulam, Hong Kong, China.; 3 The University of Hong Kong Shenzhen Institute of Research and Innovation (HKU-SIRI), 5/F, Key Laboratory Platform Building, Shenzhen Virtual University Park, No.6, Yuexing 2nd Rd, Nanshan, Shenzhen 518057, China.; 4 Faculty of Medicine & Dentistry, University of Alberta, Edmonton, AB, Canada.; 5 Department of Chemistry, Faculty of Science, The University of Hong Kong, Hong Kong, China.; 6 Department of Medicine, Li Ka Shing Faculty of Medicine, University of Hong Kong, 21 Sassoon Road, Pokfulam, Hong Kong, China.

**Keywords:** arachidonic acid metabolism, 15-keto-PGF2α, glycolysis, pyruvate kinase M2, macrophages, cardioprotection

## Abstract

Arachidonic acid (AA)-derived lipid mediators play pivotal roles in inflammation and its resolution. While glycolysis is a key metabolic pathway determining macrophage polarization, the crosstalk between specific AA metabolites and glycolytic reprogramming remains poorly understood. In this study, we explore whether certain AA metabolites modulate macrophage function through covalent protein modification, with therapeutic implications for myocardial ischemia-reperfusion injury. Unlike conventional specialized pro-resolving mediators (SPMs) that primarily act via receptors, here we identify an endogenous electrophilic AA metabolite, 15-keto-prostaglandin F2α (15KPF), that covalently modifies pyruvate kinase M2 (PKM2) at Cys49. Such interaction enhanced *PKM2* tetramerization, suppressed the *PKM2/HIF-1α/STAT3* axis, redirected energy metabolism from glycolysis to mitochondrial respiration, and promoted pro-resolving M2 macrophage polarization. Mutated *PKM2*(C49S) failed to inhibit STAT3 signaling and blocked the effect of 15KPF on M1 to M2 phenotype switch. Moreover, 15KPF reduced infarct size and preserved myocardial integrity in *in vivo* model. Taken together, covalent 15-keto-PGF2α-*PKM2* conjugation represents a self-regulatory mechanism linking AA metabolism to glycolysis to drive macrophage metabolic-inflammatory reprogramming. This pathway positions 15KPF as a promising therapeutic candidate for inflammatory and metabolic diseases, including ischemia-reperfusion injury, and distinguishes it from synthetic allosteric PKM2 activators such as TEPP-46.

## Introduction

Arachidonic acid (AA), a ω-6 polyunsaturated fatty acid, is a critical cell membrane component for maintaining structural integrity and modulating cellular functions 1. Upon cellular activation, phospholipase A2 liberates membrane-bound AA as substrate for three major enzymatic pathways: cyclooxygenase (COX), lipoxygenase (LOX), and cytochrome P450 (CYP450). These pathways generate diverse bioactive lipid mediators, including prostaglandins, leukotrienes, thromboxanes, epoxyeicosatrienoic acids (EETs), and lipoxins 2, 3. Many of these metabolites are known to regulate cell differentiation, tissue homeostasis, and organ function, and also influence the pathogenesis of aging 4, cardiovascular diseases 5, 6, hepatic fibrosis 7, neurodegeneration 8, metabolic disorders (e.g., obesity 9, diabetes 10), cancers 11, 12, and microbial infections 13. Such broad physiological and pathological roles of AA metabolites underscore the need to elucidate their mechanisms of action and therapeutic potential.

Advances in molecular biology and metabolomics have greatly facilitated the discovery of novel targets and functions for various AA metabolites 2. As examples, 15-deoxy-Δ12,14-prostaglandin J2 (15d-PGJ2) interacts with the nuclear receptor FXR to modulate cholesterol and triglyceride metabolism 14, whereas prostaglandin F2α (PGF2α) suppresses liver X receptor (LXR)-mediated transcription of cholesterol transporters (e.g., ABCA1, ABCG1) 15. Prostaglandin E2 (PGE2) exerts immunosuppressive effects via EP2/EP4 receptor signaling 16, whereas leukotrienes activate BLT1/BLT2 receptors for the recruitment of inflammatory cells in chronic diseases 17. Moreover, specialized pro-resolving mediators (SPMs)-EETs protect the heart against myocardial ischemia reperfusion injury (MIRI) by activating δ/κ-opioid receptors and mitochondrial KATP channels 18. Despite these molecular insights, the vast diversity of AA metabolites and their protein targets remains incompletely characterized.

Macrophage plasticity is central to inflammatory resolution and tissue repair but under the control of cellular metabolic reprogramming. Glycolysis, in particular, dictates the inflammatory (M1) and reparative (M2) polarization of macrophages 19. Hyperactive glycolysis fuels pro-inflammatory M1 activation, whereas suppressed glycolysis promotes anti-inflammatory M2 polarization 20, 21. Central to this metabolic reprogramming is pyruvate kinase M2 (PKM2), which functions not only as a glycolytic enzyme but also as a protein kinase and transcriptional coactivator. In its dimeric form, PKM2 translocates to the nucleus, enhances HIF-1α/STAT3 signaling, and reinforces M1 macrophage activation 22. Although synthetic PKM2 activators such as TEPP-46 promote tetramerization and suppress STAT3—thereby attenuating inflammation 23—it remains unknown whether endogenous metabolites can directly modulate PKM2 activity and macrophage polarization through covalent mechanisms. Thus, despite parallel advances in AA biology and immunometabolism, the potential crosstalk between specific lipid mediators and glycolytic pathways in macrophages represents a significant gap in our understanding of inflammatory resolution.

In this study, we bridge this gap by exploring electrophilic AA metabolites as endogenous covalent modifiers that directly reprogram macrophage function via metabolic targets. Combining click chemistry, streptavidin-based affinity enrichment, and mass spectrometry, we systematically mapped* in situ* protein targets of AA-derived electrophiles in macrophages. We identify 15-keto-PGF₂α (15KPF) as a novel endogenous ligand that covalently binds and activates PKM2—unlike conventional non-covalent synthetic activators—driving a metabolic shift from glycolysis to oxidative phosphorylation, promoting M2 polarization, and ameliorating myocardial ischemia-reperfusion injury (MIRI)* in vivo*. Our findings unveil a previously unrecognized self-regulatory mechanism linking AA metabolism to glycolytic reprogramming through covalent protein modification, offering new therapeutic avenues for inflammatory and inflammation and cardiac repair in a murine MIRI model.

## Materials and Methods

### Cell Culture

Murine macrophage cell line RAW264.7 and rat cardiomyocyte cell line H9c2 were purchased from the American Type Culture Collection (Manassas, VA, USA). Cells were maintained in Dulbecco's Modified Eagle Medium (DMEM) supplemented with 10% fetal bovine serum (FBS) and 1% penicillin/streptomycin (Thermo Fisher Scientific, USA) at 37 °C in a humidified 5% CO₂ atmosphere.

### Identification of Proteins Covalently Modified by Arachidonic Acid Metabolites

Covalent lipid-protein adducts were enriched using a Click chemistry-based enrichment protocol adapted from a published method with modifications 24. Briefly, RAW264.7 macrophages were cultured in 10-cm dishes at a density of 3 × 10⁶ cells/mL and treated with ω-alkynyl arachidonic acid (1 μg/mL) for 24 h. Cells were lysed in 0.6 mL of ice-cold RIPA buffer containing 1× proteinase inhibitor cocktail and 100 mM iodoacetamide for 30 min. Lysates were centrifuged (12,000 × g, 15 min, 4 °C), and the clarified supernatant was concentrated to 0.2 mL using a 10-kDa molecular weight cutoff centrifugal filter.

For biotinylation, concentrated proteins were reacted with a Click chemistry cocktail (100 μM azido-biotin, 1 mM CuSO₄, 100 μM Tris(benzyltriazolylmethyl)amine, 1 mM sodium ascorbate) for 4 h at room temperature. Unreacted reagents were removed by desalting via 10-kDa filtration. The biotinylated proteins were then affinity-purified using pre-washed streptavidin magnetic beads (Thermo Fisher Scientific, USA) with sequential incubation at room temperature (1 h) and 4 °C (3 h). Beads were magnetically separated, and unbound fractions (flow-through, FT) were collected. Bound proteins were washed five times (1 mL/wash) and eluted by boiling in sodium dodecyl sulfate-polyacrylamide Gel electrophoresis (SDS-PAGE) loading buffer (99°C, 5 min) to yield the eluate (E).

Protein samples (FT, W1-W5, E) were resolved by 10% SDS-PAGE. Gels were either stained with Coomassie Brilliant Blue R-250 or electroblotted onto Pierce polyvinylidene difluoride (PVDF) membranes. Membranes were probed with streptavidin-HRP or anti-PKM2 antibody, followed by visualization using enhanced chemiluminescence (ECL, GE Healthcare, Sweden).

### Proteomic Identification via MALDI-TOF-MS

Protein bands were excised and digested with trypsin (20 μg/mL) as described 24,25. Peptides were extracted in 50% acetonitrile/5% formic acid, dried, and reconstituted in 0.1% formic acid. Samples were mixed with α-cyano-4-hydroxycinnamic acid matrix (10 mg/mL in 50% acetonitrile/0.1% formic acid) and spotted onto a MALDI target plate. Peptide mapping was performed on an ABI 4800 MALDI-TOF/TOF mass spectrometer (Applied Biosystems, USA) in positive reflector mode (900-4000 Da mass range, 200 laser shots). MS/MS fragmentation (CID-MS, 2 kV) was conducted for the top five monoisotopic peaks (S/N > 200). Data were analyzed using MASCOT (NCBInr database) and GPS Explorer™ (Applied Biosystems, USA) with parameters: *Rattus norvegicus*, trypsin digestion (≤ 1 missed cleavage), carbamidomethylation, methionine oxidation, and 75 ppm mass tolerance. Scores with *p* < 0.05 were considered significant; low-scoring samples underwent further analysis.

### Isolation and Profiling of Arachidonic Acid Metabolites

Lipids from RAW264.7 cells and their culture media were extracted using 15% methanol containing 0.5% BHT. Following centrifugation (5000 rpm, 4 °C, 10 min), the supernatants were carefully acidified to pH 3 and loaded onto C18 solid-phase extraction (SPE) columns (Cayman Chemical, USA). The lipids were then efficiently eluted with 12 ml of methyl formate, gently dried under a stream of nitrogen gas, and meticulously reconstituted in 70% ethanol. Separation was achieved on a Synergi Hydro-RP C18 column (150 mm × 4.6 mm, 4 μm, Phenomenex, USA) under the control of an HPLC system (Waters Corporation, USA). A precise gradient employed acetonitrile (mobile phase A) against water (mobile phase B): 15-40% A (0-3 min), 40-50% A (3-5.5 min), isocratic 50% A (5.5-8 min), 50-70% A (8-9 min), isocratic 70% A (9-12 min), 70-15% A (12-14 min), and finally isocratic 15% A (14-15 min). The flow rate remained constant at 0.8 ml/min. Resulting fractions were assessed for covalent conjugation by incubating with RAW264.7 lysates (4 °C, overnight), followed by rigorous analysis via Click chemistry, 10% SDS-PAGE, and PKM2 immunoblotting.

### LC/MS/MS Characterization of Active AA Metabolites

Active fraction F3 was analyzed using an ABI/Sciex 3200 QTRAP LC/MS/MS system (Applied Biosystems, Canada). Lipids (20 μL) were separated on a Synergi Hydro-RP C18 column (Phenomenex, USA) under the control of Agilent 1100 HPLC system (Agilent, USA) with mobile phases: (A) 0.05% formic acid/water and (B) 0.05% formic acid/acetonitrile (0.7 mL/min, 30 °C). MS parameters were set as ESI-Turbo V source, negative ionization (-4.5 kV), 325 °C drying gas, and multiple reaction monitoring (MRM) mode (Analyst v1.4.2 software) in Table S1. The method was modified according to the previous study 26, 27.

### Mapping of 15KPF-Modified Peptides

Recombinant mouse PKM2 (20 μg) was incubated with 15KPF (2.5 μg) in PBS (37 °C, 1 h; RT, 1 h). Proteins were resolved via 10% SDS-PAGE, excised, destained, reduced with 10 mM TCEP, alkylated with 55 mM iodoacetamide, and digested with trypsin as previously described 28. Peptides were desalted with C-18 tips, dried, and analyzed via Dionex 3000 RSLCnano system (Thermo Fisher Scientific, USA) coupled to an Orbitrap Fusion Lumos mass spectrometer (Thermo Fisher Scientific, USA). Data was processed using Proteome Discoverer 2.1 (Uniprot database, 0.1% FDR). Modifications by 352.22497 Da for the formula C20H32O5 were mapped to Cys, His, or Lys residues.

### Quantitative Reverse Transcription Polymerase Chain Reaction (RT-qPCR)

Total RNA from 15KPF-treated cells was isolated with Trizol and reverse-transcribed using a RevertAid RT kit. qPCR was performed with SYBR Green (Qiagen, Germany) and primers for IL-6, iNOS, IL-1β, TNF-α, CCL2, CXCL10, IL-10, Arg1, and GAPDH. mRNA levels were normalized to GAPDH.

### Fractionation of Subcellular Proteins

Cytosolic and nuclear proteins were isolated using NE-PER reagents (Thermo Fisher Scientific, USA). Cells were lysed in CER I and CER II buffers, centrifuged (16,000 × g, 5 min), and the supernatants were collected as cytoplasmic extract. Pellets were resuspended in NER buffer, vortexed (40 min), and centrifuged (16,000 × g, 10 min) to obtain nuclear extracts. Protein concentrations were quantified as described 29.

### Western Blotting

Proteins were resolved by 10% SDS-PAGE, transferred to PVDF membranes (EMD Millipore, USA), blocked with 5% non-fat milk, and probed with primary/secondary antibodies. Protein bands were detected using ECL reagents (GE Healthcare, Sweden).

### PKM2 Cross-Linking

Following the treatment with 15KPF, RAW264.7 macrophages were lysed and incubated with disuccinimidyl suberate (2.5 mM, 30 min). Cross-linked proteins were analyzed via 10% SDS-PAGE and PKM2 immunoblotting.

### Molecular Docking

The 3D structure of 15KPF (PubChem ID: 5280887) was generated using the NCI CACTUS server. PKM2 (PDB:4B2D) was docked by AutoDock 4.2.1 (http://autodock.scripps.edu/) with 40 × 40 × 40 Å grid and Lamarckian algorithm. Results were visualized using LigPlus (http://www.ebi.ac.uk/) and PyMOL (https://pymol.org/).

### Recombinant PKM2 Production

Mouse PKM2 cDNA was cloned into bacterial expression vector pET-28a (EcoRI/XhoI). The C49S mutant was generated via site-directed mutagenesis (Vazyme, Nanjing, China) using forward primer: 5'-CATTTCTACCATTGGGCCTGCTTCCCGATCTG-3', and reverse primer: 5'-GCCCAATGGTAGAAATGATGCCAGTGTTGCGG-3'). Plasmids were transformed into *E. coli* BL21(DE3), induced with 0.5 mM IPTG (30 °C, 6 h), and purified via HisTrap™ HP columns under the control of ÄKTAexplorer chromatography system (GE Healthcare, Sweden).

### Isothermal Titration Calorimetry (ITC) Assay

Recombinant PKM2, PKM2(C49S), and 15KPF were dissolved in PBS (pH 7.2). Titrations (75 μM protein into 200 μM 15KPF) were performed using a MicroCal PEAQ-ITC system. Data were analyzed with a single-site binding model.

### Cellular Thermal Shift Assay (CETSA)

Cell lysates were treated with 0.5 mg/ml of 15KPF or a vehicle control. The samples were then aliquoted and heated using a thermal cycler at a precise temperature gradient ranging from 40 to 75 °C for 5 minutes. After heating and subsequent cooling, the lysates were centrifuged at high speed to separate the soluble, stabilized proteins from the precipitated, denatured proteins. The resulting supernatants were finally analyzed by Western blotting.

### Drug Affinity Responsive Target Stability (DARTS)

Cell lysates were treated with the indicated doses of 15KPF or control for binding. The mixture was then subjected to proteolysis by adding Pronase at a 1:250 dilution. The reaction was stopped by adding protease inhibitors, and the samples were analyzed by Western blotting.

### Seahorse Assay

RAW264.7 cells (10,000/well) were treated with 15KPF/LPS (12 h). ECAR and OCR were respectively measured by Glycolysis Stress Kit and Mito Stress Kit on a Seahorse XFe96 Analyzer (Agilent, USA). Data was normalized to cell counts.

### Lentiviral PKM2 Expression/Silencing

PKM2 WT/C49S were cloned into pCDH-CMV (IGE Biotechnology, Guangzhou, China). Lentivirus was produced in 293FT cells using packaging plasmids (pRSV-Rev, pMDLg/Prre, pMD2.G). RAW264.7 cells were transduced (48 h) and selected with puromycin. For gene silencing, PKM2 shRNA (sequence: 5′-CCGGGCGGTGGCTCTGGATACAAAGCTCGAGCTTTGTATCCAGAGCCACCGCTTTTTGAATT-3′) was delivered via pLKO.1-CMV.

### Myocardial Ischemia-Reperfusion Injury Model

The animal protocols adhered to Directive 2010/63/EU and were approved by the University of Hong Kong (CULATR 4899-18). Male C57BL/6N mice, weighing between 23-28 grams, underwent left anterior descending (LAD) artery ligation, resulting in 45 minutes of ischemia, followed by reperfusion for 3, 5, or 7 days. Sham-operated mice underwent surgery without ligation. The study included three groups (n = 10/group): Sham, Model, and 15KPF. Mice in the Sham group (n = 10) received a vehicle at a dose of 0.5 mg/kg every 12 hours via *intraperitoneal (*i.p.) injection for 3, 5, and 7 days. Mice with LAD artery ligation were randomly assigned to either the Model group (n = 10), which underwent reperfusion for 3, 5, or 7 days and received vehicle via i.p. injection, or the 15KPF group (n = 10), which also underwent reperfusion for 3, 5, or 7 days and received 15KPF at 0.5 mg/kg every 12 hours via i.p. injection for the same duration. The initial dose of 15KPF or vehicle was administered before the reperfusion began. The dose of 0.5 mg/kg was chosen based on preliminary dose-ranging experiments and literature supporting the efficacy of a similar compound, 15d-PGJ2, within this dosage range 29.

### Infarct Size and Histopathology

Infarct area was visualized by TTC staining while histology was examined by H&E staining, CD68/CD206 immunohistochemistry (IHC) as described 30. Images were analyzed using ImageJ.

### Statistical Analysis

Data was expressed as mean ± SD/SEM and analyzed by One-way ANOVA with Dunnett's test (GraphPad Prism 5), *p* < 0.05 was significant.

## Results

### Covalent Modification of PKM2 by 15-Keto-PGF2α (15KPF)

**Figure 1:**
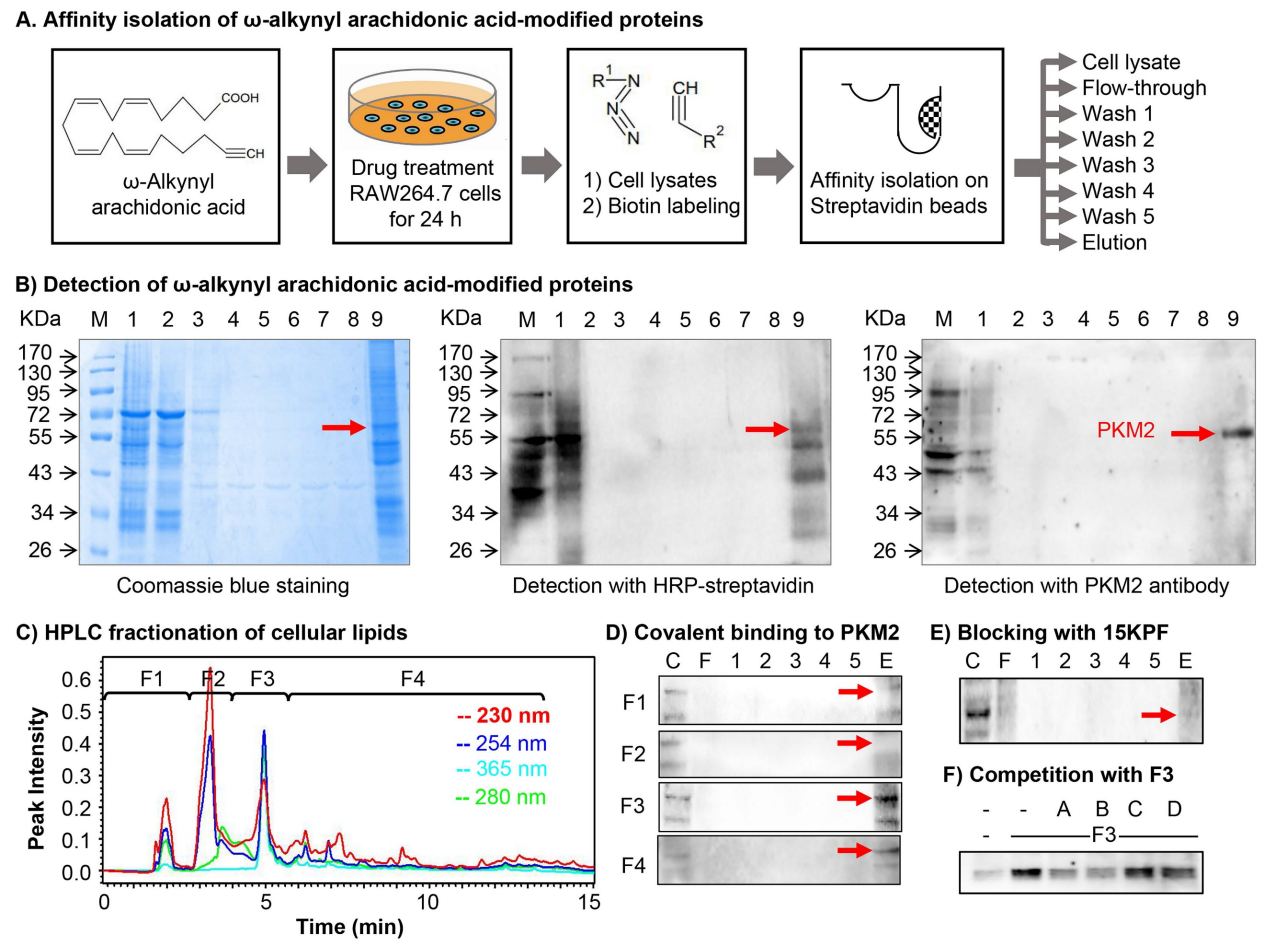
** Molecular identification of the covalent conjugates between arachidonic acid metabolites and cellular proteins.** (A) Scheme for isolating arachidonic acid metabolite-modified proteins. After the treatment with 1 μg/mL of ω-alkynyl arachidonic acid for 24 h, the cellular proteins were extracted and labelled by Click chemistry biotinylation. The reaction products were separated into different fractions with streptavidin-coated magnetic beads. (B) Coomassie blue detection of arachidonic acid metabolite-modified proteins. The protein fractions were separated by 10% SDS-PAGE and visualized with Coomassie Brilliant Blue R-250 or detected by Western blot analysis with either streptavidin-HRP conjugate or anti-PKM2 antibody/HRP-conjugated secondary antibody. (C) HPLC fractionation of macrophage lipids. RAW264.7 cells were incubated with ω-alkynyl arachidonic acid while the cellular lipids were extracted and fractionated into 4 fractions on a HPLC system. (D) Assay of arachidonic acid metabolites for covalent binding to PKM2. Following overnight incubation of macrophage lysates with 4 cellular lipid fractions, the reaction products were biotinylated, purified with streptavidin-coated magnetic beads and detected by Western blot analysis with anti-PKM2 antibody. (E) Effects of 15KPF on the covalent conjugation of ω-alkynyl arachidonic acid metabolites with PKM2. RAW264.7 lysates were sequentially incubated with 15KPF for 3 h and HPLC fraction F3 overnight. The reaction products were separated by 10% SDS-PAGE and detected by Western blot analysis with anti-PKM2 antibody. (F) Evaluation of four 15KPF analogues for competitive binding to PKM2. RAW264.7 lysates were sequentially incubated with four analogues for 3 h and HPLC fraction F3 overnight. The reaction products were separated by 10% SDS-PAGE and blotted with anti-PKM2 antibody. A, 15KPF; B, 13,14-dihydro-15-keto-PGF2α; C, PGF2α; D, 15-HETE.

To identify arachidonic acid (AA) metabolites capable of covalently conjugating with cellular proteins in macrophages and characterize their biological implications. Macrophage RAW264.7 cells were treated with ω-alkynyl AA for metabolic labeling. Biotinylated proteins were enriched via Click chemistry and streptavidin pulldown as outlined in Figure 1A. As shown in Figure 1B, SDS-PAGE and Coomassie staining revealed a prominent band at 55-60 kDa, while proteomic identification (MALDI-TOF-MS) and Western blotting confirmed PKM2 as the predominant target. On the other hand, the active AA metabolites were profiled by two strategies: (1) lipid fractionation and (2) competitive binding bioassays with AA metabolites. As a result, lipid fractions (F1-F4) (Figure 1C) from HPLC separation were tested for PKM2 binding. Fraction F3 showed strong interaction (Figure 1D), and LC/MS/MS analysis subsequently identified 15KPF as the dominant component (Figure 1E). Pre-incubation with 15KPF or its derivative 13,14-di-15-keto-PGF2α blocked F3-PKM2 conjugation (Fig. 1F). These results indicate that 15KPF covalently modifies PKM2, suggesting its role as a key regulator of PKM2 activity.

### Validation of 15KPF-PKM2 Interaction

**Figure 2:**
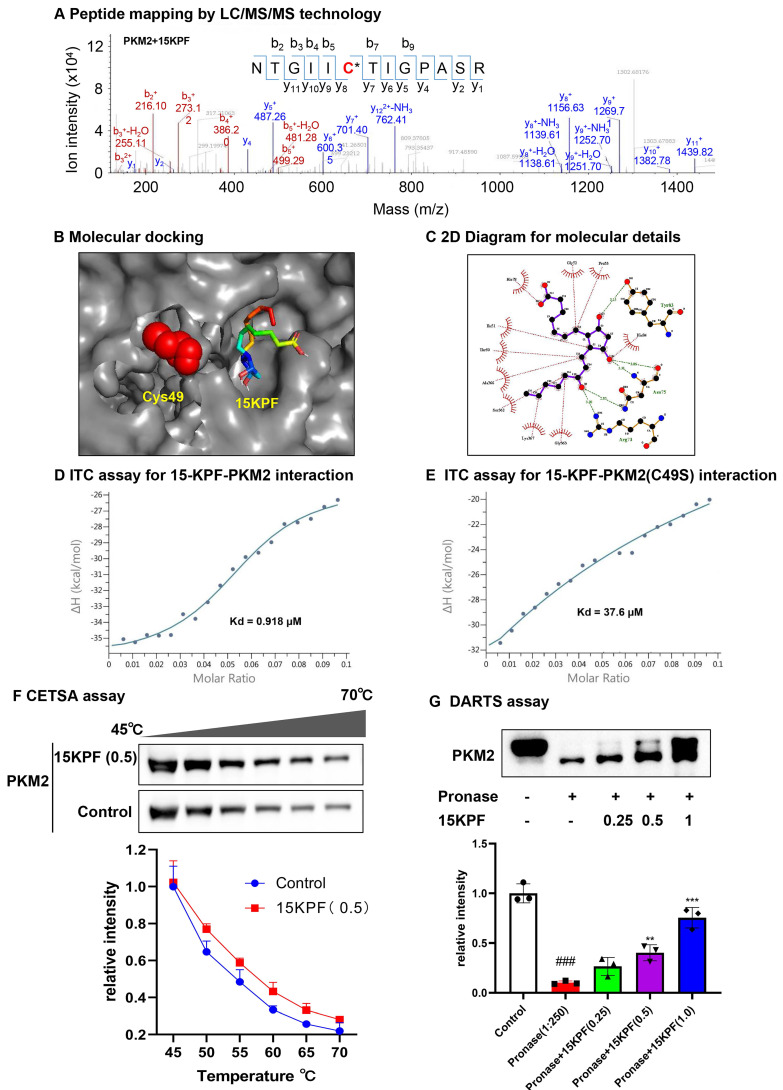
** Covalent conjugation of PKM2 with 15KPF.** (A) Mapping of 15KPF-binding site in PKM2. After incubation with vehicle or 15KPF, recombinant PKM2 was digested with trypsin. Peptides were mapped by LC/MS/MS technology as described in “Methods”. 15KPF-modified peptides were identified by the increase in the mass of peptide ions. (B) Molecular docking of 15KPF to PKM2. 15KPF (PubChem ID: 5280887) and 3D structure of PKM2 (PDB ID: 4B2D) were analyzed. (C) Diagram illustrating the 2D 15KPF-PKM2 interactions. The docking results were converted to 2D plot by Ligplot^+^ (http://ebi.ac.uk) Red dotted lines indicate hydrophobic interaction whereas green dotted lines indicate hydrogen bond. (D) ITC assay for the 15KPF-PKM2 interaction. PKM2 was injected into the sample cell containing 15KPF. (E) ITC assay for the 15KPF-PKM2(C49S) interaction. PKM2(C49S) was injected into the sample cell containing 15KPF. (F) CETSA assay showed that 15KPF protected PKM2 at different temperature gradients in RAW264.7 cells. n = 3 from 3 independent experiments; (G) DARTS assay showed that 15KPF promoted the resistance of PKM2 to pronase digestion in RAW264.7 cells. n = 3 from 3 independent experiments. The data were expressed as means ± SD and analyzed by one-way ANOVA, followed by Dunnett's multiple comparisons test. Control vs pronase, ###, p < 0.001; Pronase vs 15KPF treatment, **, p < 0.01, ***, p < 0.001.

To validate the covalent interaction between 15KPF and PKM2, recombinant PKM2 was incubated with 15KPF, and peptide mapping (LC/MS/MS) was performed. LC/MS/MS results revealed that the peptide NTGII**C^49^**TIGPASR appeared to have a mass shift (+352.22 Da), confirming covalent attachment at Cys^49^ (Figure 2A). Molecular docking (Autodock Vina) and isothermal titration calorimetry (ITC) were used to analyze binding. Molecular docking positioned 15KPF near Cys^49^, forming hydrogen bonds with Arg^73^, Asn^75^, and Tyr^83^, and van der Waals interactions with Thr^50^ and Ile^51^ (Figure 2B-C). ITC confirmed high-affinity binding (Kd = 0.918 μM) to wild-type PKM2, but not PKM2(C49S) (Kd = 37.6 μM) (Figure 2D-E). Additionally, we utilized CETSA and DARTS assays to confirm PKM2 as the target of 15KPF. Figure 2F illustrates that the band corresponding to PKM2 nearly vanished completely in control cells between 40 °C and 75 °C, whereas the band remained in cells treated with 15KPF. The DARTS results (Figure 2G) further demonstrated that 15KPF significantly enhanced PKM2 stabilization against pronase-induced degradation. These outcomes suggest that 15KPF forms a covalent bond with PKM2 at Cys49, providing a structural foundation for functional modulation.

### 15KPF Modulates Macrophage Polarization and Phagocytosis

**Figure 3:**
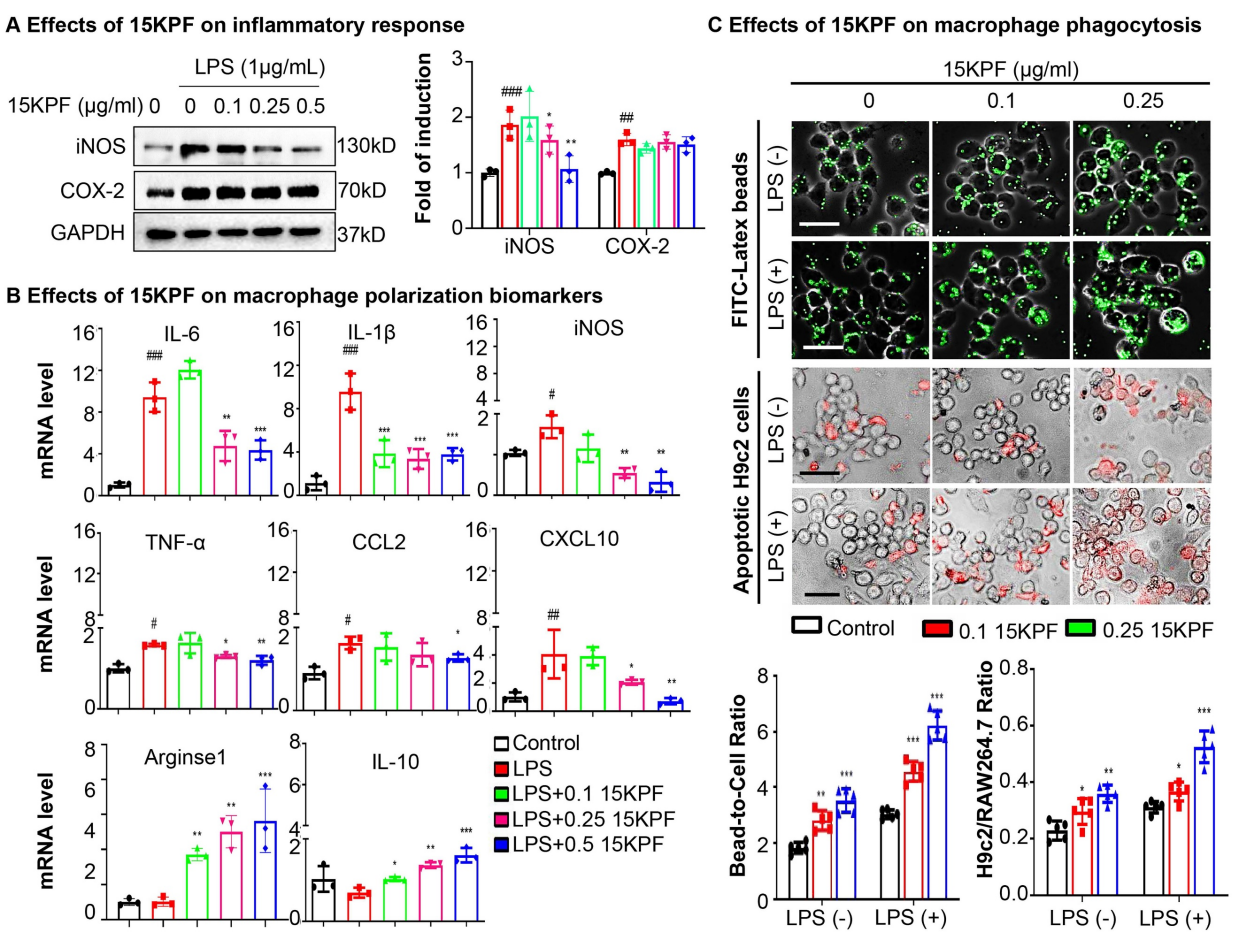
*** In vitro* effects of 15KPF on the phenotypes and phagocytic activity of macrophages.** (A) Western blot analysis of COX-2 and iNOS. Following the treatment with 15KPF and LPS, RAW264.7 macrophages were lysed and examined by Western blotting with antibodies against COX-2 and iNOS. n = 3 from 3 independent experiments. The data were expressed as means ± SD and analyzed by one-way ANOVA, followed by Dunnett's multiple comparisons test. Control vs LPS, ##, p<0.01, ###, p<0.001; LPS vs 15KPF treatment, *, p<0.05; **, p<0.01. (B) qRT-PCR determination for the mRNA levels of macrophage M1 and M2 biomarkers. The total RNAs were isolated for quantitative analysis of specific biomarkers by qRT-PCR technique. n = 3 from 3 independent experiments. The data were expressed as means ± SD and analyzed by one-way ANOVA, followed by Dunnett's multiple comparisons test. Control vs LPS, #p<0.05, ##, p<0.01, ###, p<0.001; LPS vs 15KPF treatment, *, p<0.05; **, p<0.01, ***, p<0.001. (C) Effect of 15KPF on the phagocytic activity of macrophages. RAW264.7 macrophages were treated with 15KPF and LPS, alone or in combination, for 24 h, and then allowed to uptake FITC-labeled beads or apoptotic H9c2 cells that were labelled with Cell Tracker Orange CMTMR dye. The cells were imaged on a fluorescence microscope. The images were quantified with NIH ImageJ software. n = 5 from 3 independent experiments. The data were expressed as means ± SD and analyzed by one-way ANOVA, followed by Dunnett's multiple comparisons test. *, p<0.05; **, p<0.01, ***, p<0.001. Scale bar, 50 μm.

To determine the effects of 15KPF on macrophage polarization and phagocytosis, RAW264.7 cells were treated with LPS ± 15KPF. M1/M2 biomarkers (iNOS, COX-2, IL-6, TNF-α, Arg1, IL-10) were assessed via immunoblotting and qRT-PCR. LPS-stimulated RAW264.7 macrophages exhibited elevated M1 markers (iNOS, IL-6, TNF-α) and suppressed M2 markers (Arg1, IL-10). 15KPF treatment suppressed LPS-induced M1 markers (iNOS, IL-6, TNF-α) and upregulated M2 markers (Arg1, IL-10) (Figure 3A-B). Phagocytosis was quantified using fluorescent beads/apoptotic cardiomyocytes. 15KPF enhanced phagocytosis of latex beads and apoptotic H9c2 cardiomyocytes even under LPS stimulation (Figure 3C). These results indicate that 15KPF skews macrophages toward an anti-inflammatory M2 phenotype, enhancing their reparative functions.

### Mechanistic Insights into PKM2-Dependent Signaling

**Figure 4:**
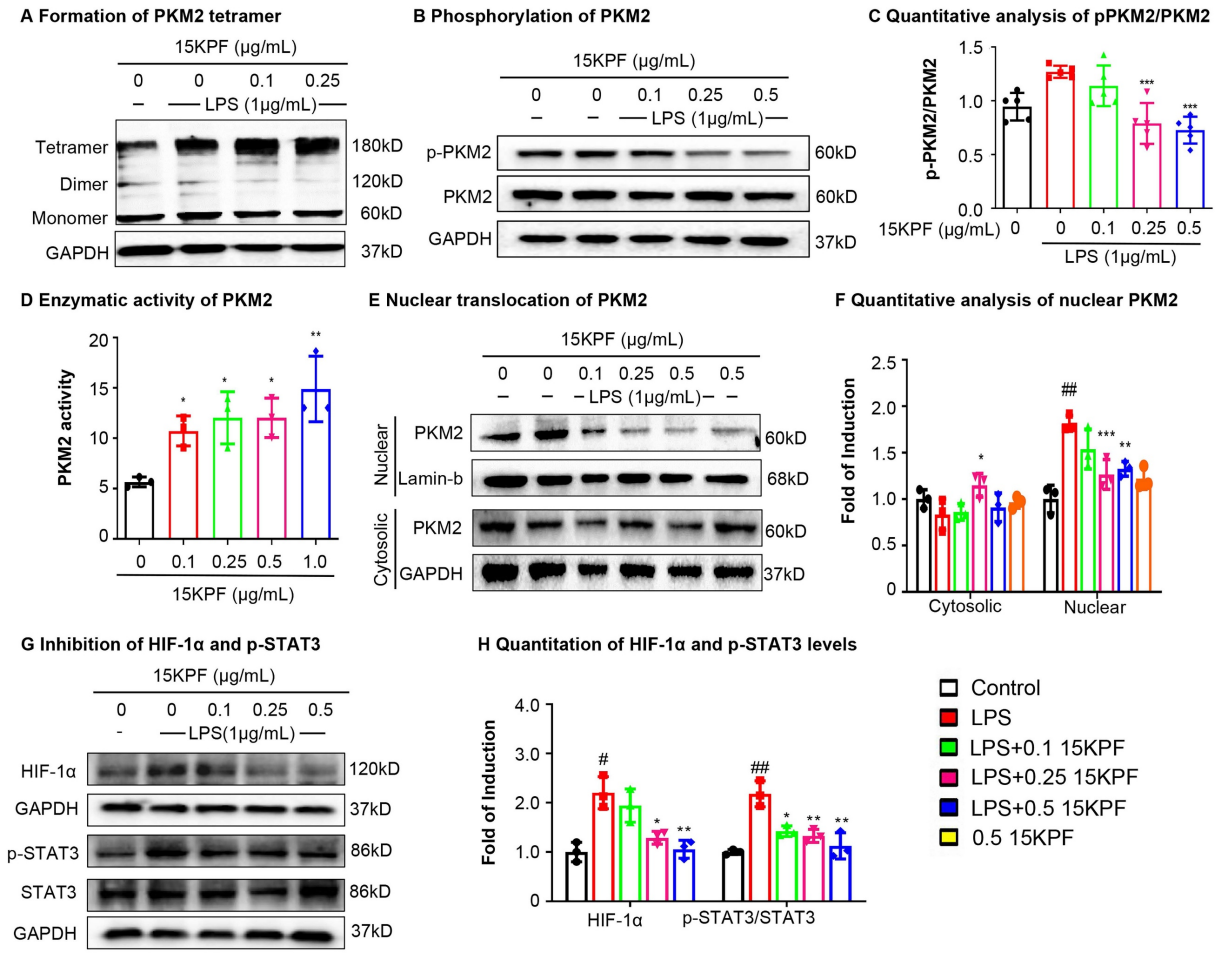
** Effects of 15KPF binding on the biological functions of PKM2.** (A) Increased formation of PKM2 tetramer. After treatment with 15KPF, the cell lysates were cross-linked with 2.5 mM disuccinimidyl suberate and blotted with anti-PKM2 antibody. (B & C) Inhibition of PKM2 phosphorylation against LPS stimulation. RAW264.7 cells were treated with 15KPF and LPS as indicated and blotted with specific antibodies. n = 5 from 5 independent experiments. The data were expressed as means ± SD and analyzed by one-way ANOVA, followed by Dunnett's multiple comparisons test. ***, p<0.001. (D) Enhanced enzyme activity of PKM2. The cells were treated with 15KPF at the indicated concentrations and assayed for PKM2 activity using commercial colorimetric assay kit. n = 3 from 3 independent experiments. The data were expressed as means ± SD and analyzed by one-way ANOVA, followed by Dunnett's multiple comparisons test. *, p < 0.05; **, p < 0.01. (E& F) Reduced nuclear translocation of PKM2. Following the treatment with 15KPF and LPS, RAW264.7 cells were lysed and fractionated into the cytosolic and nuclear proteins. The protein preparations were blotted with anti-PKM2 antibody. Lamin b indicated the loading of nuclear proteins whereas GAPDH indicated the loading of cytosolic proteins. n = 3 from 3 independent experiments. The data were expressed as means ± SD and analyzed by one-way ANOVA, followed by Dunnett's multiple comparisons test. Control vs LPS, #p<0.05, ##, p<0.01, ###, p<0.001; LPS vs 15KPF treatment, *, p < 0.05; **, p < 0.01; ***, p<0.001. (G and H) Inhibition of HIF-1α pathway and STAT3 pathway. Following the treatment with 15KPF and LPS, RAW264.7 cells were lysed and blotted with antibodies against HIF-1α, p-STAT3, and STAT3. GAPDH indicated protein loading. n = 3 from 3 independent experiments. The data were expressed as means ± SD and analyzed by one-way ANOVA, followed by Dunnett's multiple comparisons test. Control vs LPS, #p<0.05, ##, p<0.01; LPS vs 15KPF treatment, *, p<0.05, **, p<0.01.

To investigate the effects of 15KPF on PKM2-mediated signaling, experiments were performed to assess PKM2 dimer/tetramer ratios, phosphorylation (Tyr¹⁰⁵), nuclear translocation, enzyme activity and HIF-1α/STAT3 signaling. As a result, 15KPF preferably promoted the formation of PKM2 tetramer over dimer (Figure 4A), which effectively diminishes the phosphorylation of PKM2 at Tyr^105^ against LPS stimulation (Figure 4B-C). Based on Figure 4E and 4F, LPS markedly induces the nuclear translocation of PKM2, whereas 15KPF attenuates the nuclear translocation of PKM2 against LPS stimulation in a concentration-dependent fashion. Additionally, the enzyme activity of PKM2 was determined in response to 15KPF. As shown in Figure 4D, 15KPF increased PKM2 activity in a concentration dependent fashion. According to Figure 4G and 4H, LPS increases HIF-1α and p-STAT3 protein levels whereas 15KPF diminishes the protein levels of HIF-1α and p-STAT3 against LPS. These results suggest that 15KPF may inhibit pro-inflammatory HIF-1α/STAT3 pathways via PKM2 targeting.

### Metabolic Reprogramming by 15KPF

**Figure 5:**
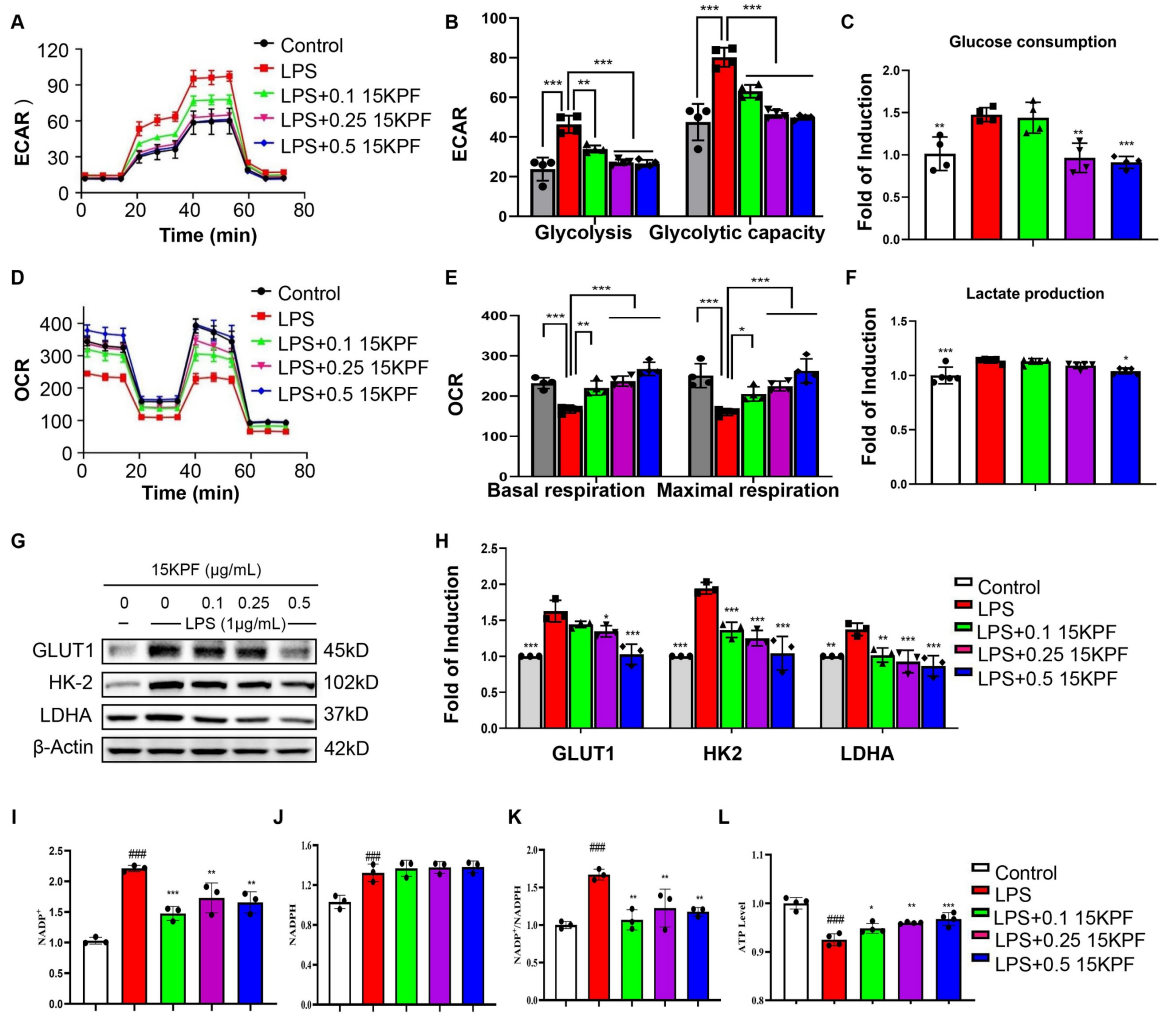
** Effects of15KPF on glycolysis and mitochondrial respiration in LPS-activated macrophages.** (A) ECAR assay. ECAR was determined as described in “Methods”. (B) Basic and maximal capacity of glycolysis. n = 4 from 4 independent experiments. (C) Determination of glucose consumption. n = 5 from 5 independent experiments. After drug treatment, the cellular glucose was measured with the commercial kit. (D) OCR assay. OCR was determined as described in “Methods”. (E) Basic and maximal capacity of mitochondrial respiration. n = 4 from 4 independent experiments. (F) Determination of lactate production. After drug treatment, the cellular lactate was measured with the commercial kit. n = 5 from 5 independent experiments. (G-H) Western blot analysis of key glycolytic proteins. n = 3 from 3 independent experiments. The data were expressed as means ± SD and analyzed by one-way ANOVA, followed by Tukey test. *, p < 0.05; **, p < 0.01; ***, p < 0.001. (I-L) The effect of 15KPF on the levels of NADP+, NADPH, NADP+/NADPH and ATP in LPS-induced RAW264.7 macrophages. n = 3 or 4 from 3 or 4 independent experiments. The data were expressed as means ± SD and analyzed by one-way ANOVA, followed by Dunnett's multiple comparisons test. Control vs LPS, ###, p<0.001; LPS vs 15KPF treatment, *, p < 0.05, **, p < 0.01, ***, p < 0.001.

To investigate the impact of 15KPF on the macrophage metabolic pathways, the glycolytic and mitochondrial functions of RAW264.7 cells were assessed by using Agilent Seahorse XF Analyzer. Figure 5A and 5B show that whereas 15KPF antagonistically counteracted the effects of LPS on glycolysis in a concentration-dependent manner. Concurrently, 15KPF restored the mitochondrial respiratory capacity that was impaired by LPS (Figure 5D and 5E), indicating a shift from glycolytic metabolism toward oxidative phosphorylation. Consistent with these observations, 15KPF reduced glucose uptake and lactate production in LPS-stimulated macrophages (Figure 5C and 5F), further supporting its inhibitory effect on glycolytic flux. To elucidate the molecular basis of this metabolic rewiring, we examined the expression of key glycolytic enzymes. Western blot analysis revealed that LPS markedly up-regulated GLUT1, HK-2, and LDHA, and this induction was reversed by 15KPF treatment (Figure 5G and 5H). Importantly, 15KPF also enhanced the NADP+/NADPH ratio and increased ATP production (Figure 5I-5L), suggesting a reinforcement of mitochondrial energy metabolism and redox homeostasis. The elevated NADP+/NADPH ratio implies enhanced flux through pathways such as the pentose phosphate pathway, which supports antioxidant capacity and biosynthetic precursors. Meanwhile, the increase in ATP output reflects efficient coupling between the TCA cycle and oxidative phosphorylation, underscoring a restored mitochondrial metabolic phenotype. Metabolomics analysis revealed 15KPF regulated TCA product oxoglutaric acid and oxalacetic acid in supplementary Figure S1. Together, these results demonstrate that 15KPF not only suppresses glycolysis but also promotes mitochondrial bioenergetics by rebalancing NADP+/NADPH redox status and enhancing ATP generation through increased TCA cycle activity and respiratory chain function.

### PKM2-Dependent Regulation of STAT3 and Polarization

**Figure 6:**
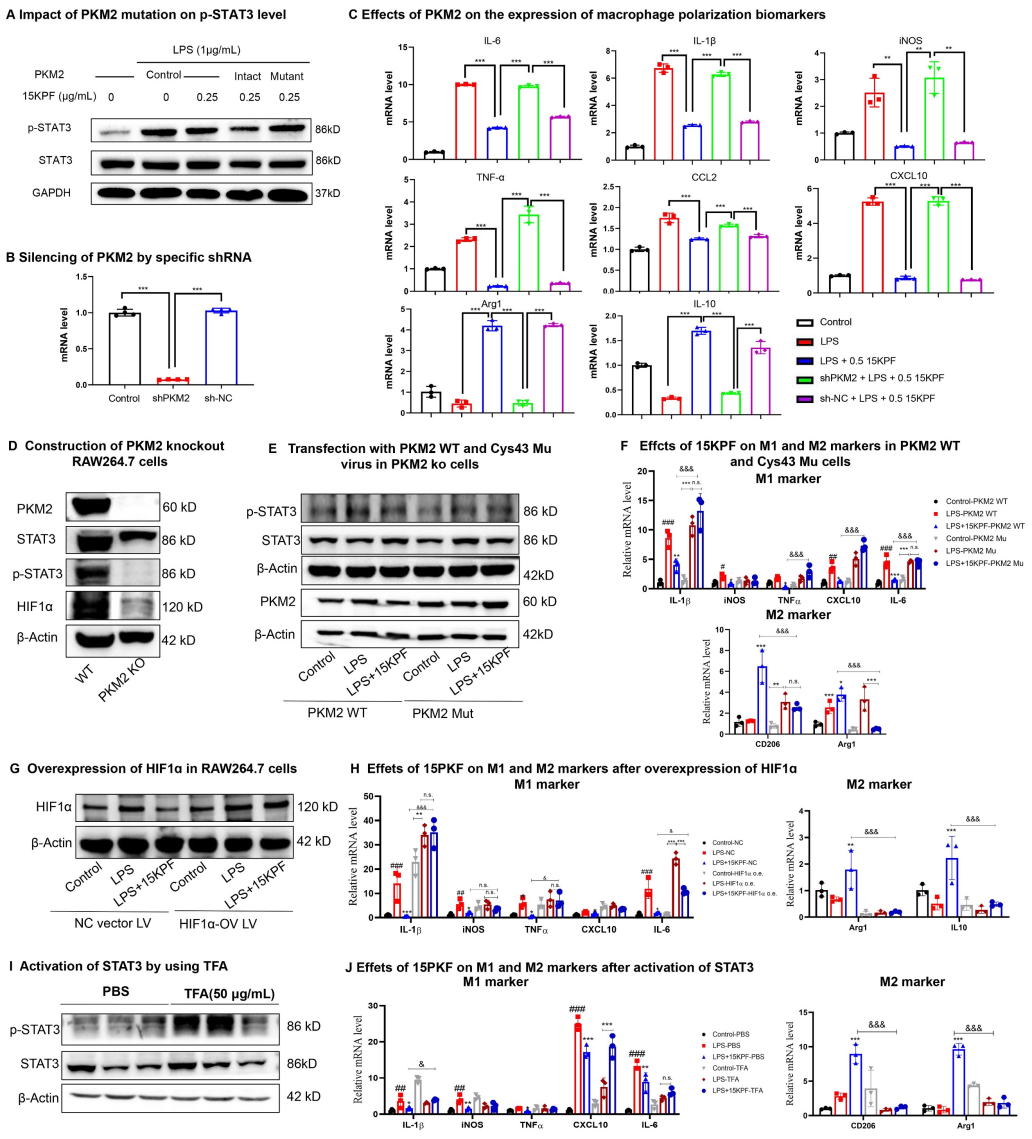
** Essential role of intact PKM2 in mediating the biological activities of 15KPF.** (A) C49 of PKM2 is essential for 15KPF to inhibit STAT3. After transfection with PKM2 or PKM2(C49S), RAW264.7 cells were treated with 15KPF, lysed and analyzed with antibody against STAT3 or p-STAT3. The results were from three independent experiments. (B) Silencing of PKM2 by specific shRNA. RAW264.7 cells were transfected with shPKM2 or sh-NC (vector alone). PKM2 mRNA was quantified by qRT-PCR technique. n = 4 from 4 independent experiments. ***, p<0.001. (C) Effects of PKM2 on the expression of macrophage polarization biomarkers. Following the treatment with 15KPF and LPS, RAW264.7 cells were subjected to the extraction of total RNAs and qRT-PCR quantification of macrophage biomarkers with specific primers. n = 4 from 4 independent experiments. The data were expressed as means ± SD and analyzed by one-way ANOVA, followed by Dunnett's test. **, p<0.01, ***, p<0.001. (D) Construction of PKM2 knockout RAW264.7 cells. The results were from three independent experiments. (E) The effect of 15KPF on p-Stat3 after transfection with PKM2 WT and C49s Mutant in PKM2 KO cells. The results were from three independent experiments. (F) Effect of 15KPF on M1 and M2 markers in PKM2 WT and C49S mutant cells. n = 3 from 3 independent experiments. The data were expressed as means ± SD and analyzed by one-way ANOVA, followed by Dunnett's test. #, p<0.05, ##, p<0.01, ###, p<0.001, LPS vs control group; *, p<0.05, **, p<0.01, ***, p<0.001, 15KPF+LPS vs LPS group; &&&, p<0.001, 15KPF+LPS in PKM2 C49S mutant vs 15KPF+LPS in PKM2 WT group. (G) Overexpression of HIF1ɑ in RAW264.7 cells. The results were from three independent experiments. (H) Effect of 15KPF on M1 and M2 markers after HIF1ɑ overexpression. n = 3 from 3 independent experiments. #, p<0.05, ##, p<0.01, ###, p<0.001, LPS vs control group; *, p<0.05, **, p<0.01, ***, p<0.001, 15KPF+LPS vs LPS group; &&&, p<0.001, 15KPF+LPS in NC vs 15KPF+LPS in HIF1ɑ o.v. group. (I) Activation of STAT3 by using TFA. The results were from three independent experiments. (J) Effect of 15KPF on M1 and M2 markers after STAT3 activation. n = 3 from 3 independent experiments. ##, p<0.01, ###, p<0.001, LPS vs control group; *, p<0.05, **, p<0.01, ***, p<0.001, 15KPF+LPS vs LPS group; &, p<0.05, &&&, p<0.001, 15KPF+LPS in TFA vs 15KPF+LPS in PBS group.

To clarify the importance of covalent 15KPF-PKM2 conjugation, intact PKM2 cDNA and PKM2 (C49S) cDNA were cloned. They were then inserted into lentivirus expression vectors to yield pCDH-CMV-PKM2 plasmid or pCDH-CMV-PKM2(C49S) plasmid and introduced into RAW264.7 cells. The transfected RAW264.7 cells were treated with 15KPF at different concentrations and analyzed for p-STAT3 levels. As shown in Figure 6A, 15KPF reduces the level of p-STAT3 in PKM2 transfected cells but fails to inhibit STAT3 phosphorylation in PKM2(C49S)-transfected cells. Figure 6B shows that PKM2 expression was successfully silenced by PKM2 shRNA plasmid (sh-PKM2). The results in Figure 6C showed that PKM2 silence reversed the effects of 15KPF on the expression of macrophage biomarkers in LPS-treated RAW264.7 cells. To examine the response of PKM2 to 15KPF, a PKM2 knockout RAW264.7 macrophage cell line was constructed (Figure 6D) while PKM2 WT and C49S mutant lentiviral particles were prepared. Subsequently, PKM2 knockout RAW264.7 macrophages were infected with PKM2 WT and C49S mutant virus (Figure 6E). The effects of 15KPF on M1 and M2 markers were further measured. As shown in Figure 6F, 15KPF suppressed M1 markers while upregulating M2 markers in PKM2 WT cells. By contrast, such effects were hardly detected in PKM2 C49S mutant cells. We also prepared HIF1-ɑ lentivirus and overexpressed HIF1-ɑ in RAW264.7 macrophages by lentivirus infection (Figure 6G). Indeed, we found that HIF1-ɑ overexpression attenuates the inhibitory effect of 15KPF on M1 markers and the elevated effect of M2 markers (Figure 6H). As for mimicking constitutive activation of STAT3 in cells, we used STAT3 activator Colivelin (TFA) in the assay (Figure 6I). We found that STAT3 activation blocks the stimulatory effect of 15KPF on M2 markers and the inhibitory effects of 15KPF on M1 makers, such as IL-1β, CXCL10 and IL-6 (Figure 6J). These results confirm that 15KPF modulates STAT3 signaling and macrophage polarization through covalent PKM2-Cys⁴⁹ interactions.

### Cardioprotective Effects of 15KPF *In Vivo*

**Figure 7:**
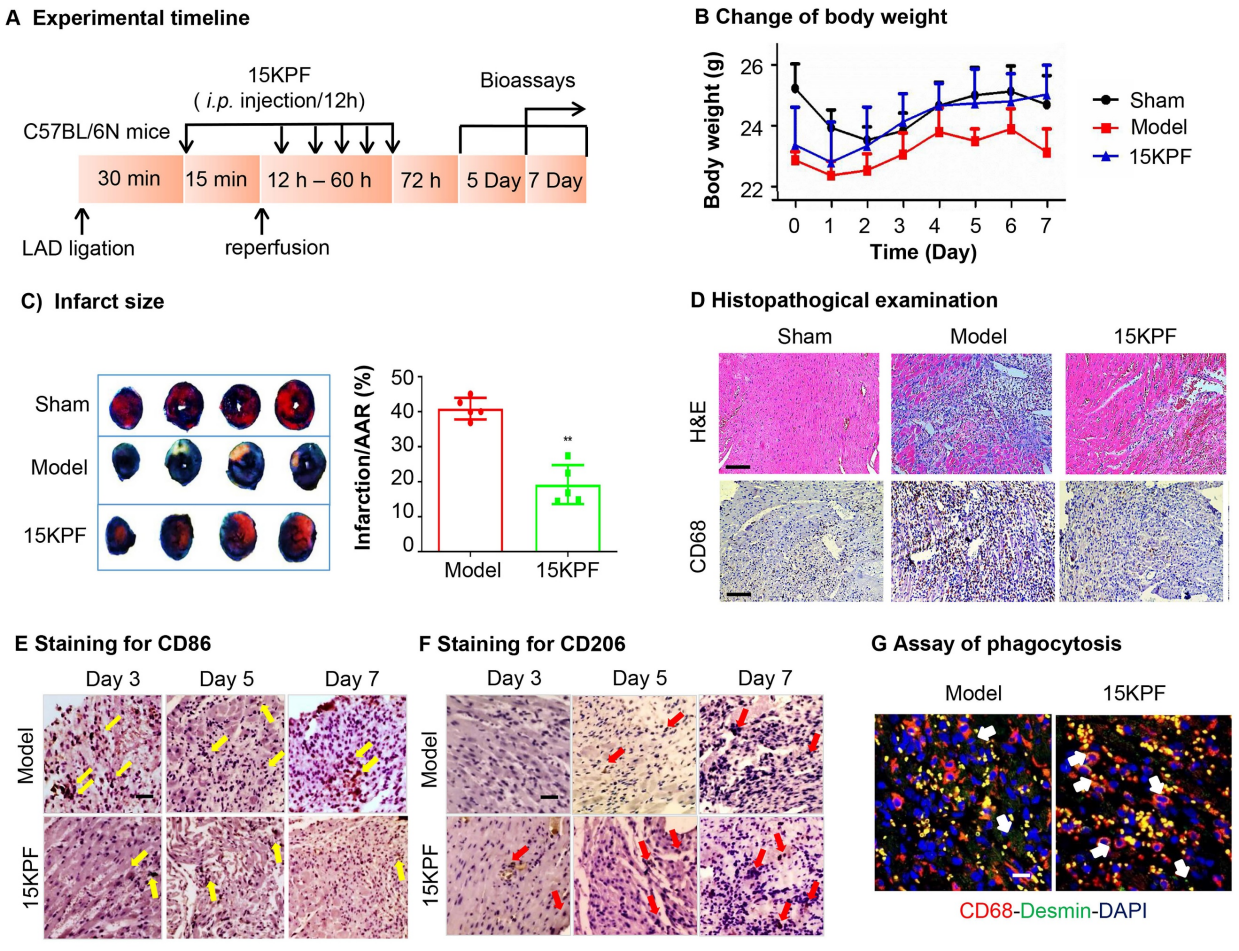
***In vivo* effects of 15KPF on macrophage polarization against acute myocardial infarction.** (A) Design of animal experiments. C57BL/6N mice were randomly divided into three experimental groups (n = 10 from 3 independent experiments): Sham, Model, 15KPF (0.5 mg/kg/12 h). Following surgery, mice received vehicle as Model group or 15KPF as 15KPF group. The animals were euthanized on Day 3, 5 and 7 whereas the hearts were collected for examinations. (B) Monitoring of body weight. Body weight was regularly monitored. n = 8 from 3 independent experiments). (C) Assessment of infarct size. Following ischemia and reperfusion, the cardiac tissues were subjected to TTC staining and imaged under a microscope. Infarct sizes and the area-at-risk were quantified. The data were expressed as mean ± SD and analyzed by unpaired student's t-test. n = 5 from 3 independent experiments, ***, p < 0.001. (D) Pathological assessments of cardiac tissues. The cardiac tissues were subjected to H&E staining and immunohistochemical detection of pan-macrophage biomarker CD68. The images at 400× amplification were captured under a microscope. Scale bar, 50 µm. (E) Histochemical detection of M1 macrophages. Following ischemia and reperfusion, the heart tissues were collected at Day-3, 5, or 7, and stained with anti-CD86 antibody. (F) Histochemical detection of M2 macrophages. Following ischemia and reperfusion, the heart tissues were collected at Day-3, 5, or 7, and stained with anti-CD206 antibody. (G) Detection of phagocytosis in infarcted myocardium. The hearts were collected and examined by immunostaining of CD68 and desmin with specific antibodies. Scale bar, 50 μm.

To evaluate the* in vivo* cardioprotective effects of 15KPF, myocardial ischemia-reperfusion injury (MIRI) was induced in mice. Following 15KPF treatment (0.5 mg/kg), infarct size, macrophage polarization (CD86⁺/CD206⁺), and phagocytosis were analyzed. As results, 15KPF (0.5 mg/kg) reduced infarct size by 18%, improved the bodyweight (Figure 7A-C) and preserved cardiac tissue integrity (Figure 7D). Immunofluorescence revealed that 15KPF shifted cardiac macrophages from CD86⁺ M1 to CD206⁺ M2 type (Figure 7E-F) and enhanced phagocytosis of apoptotic cardiomyocytes (Figure 7G). Additionally, BMDM cells isolated from each group showed that 15KPF significantly decreased M1 markers (iNOS, IL-1β, IL6, CCL2, CXCL10, CD86) while increased M2 markers (Arg1, PPARγ, IL10, Ym1, CD206) (Figure S2). These results indicate that 15KPF mitigates post-MI damage by promoting reparative macrophage polarization, highlighting therapeutic potential.

## Discussion

Dynamic metabolic and inflammatory reprogramming not only controls the balance between pro-inflammatory M1 and reparative M2 polarization, but also critically influences tissue damage and recovery 31-33. Emerging evidence underscores a central role of metabolic reprogramming, particularly glycolytic flux and AA metabolism, in the regulation of macrophage activation and inflammatory responses 34-36. While glycolysis fuels the pro-inflammatory M1 phenotype, mitochondrial oxidative phosphorylation supports the anti-inflammatory M2 state, offering therapeutic opportunities to modulate macrophage plasticity 37-40. Here, we identify an AA metabolite 15-keto-prostaglandin F2α (15KPF) as a novel covalent modulator of PKM2, bridging AA metabolism to glycolytic rewiring and macrophage plasticity. Our findings reveal a previously unrecognized crosstalk between AA signaling and glycolysis, with profound implications for resolving inflammation and promoting cardiac repair in post-MI hearts.

The covalent conjugation of 15KPF to PKM2 at Cys49 by a Michael addition reaction represents a key mechanistic insight. PKM2 is a glycolytic enzyme with dual metabolic and transcriptional roles, and thereby greatly influences macrophage polarization and inflammation 41-43. Under LPS stimulation, PKM2 shifts from its glycolytically active tetrameric form to phosphorylated and nuclear-translocating dimers, gaining the capability to drive HIF-1α/STAT3-dependent production of pro-inflammatory cytokines (e.g., IL-1β, IL-6) 44-46. By stabilizing PKM2 tetramers and enhancing its enzymatic activity, 15KPF suppresses the activity of LPS on phosphorylation (Y105), dimerization, and nuclear translocation of PKM2. Such inhibition disrupts the PKM2-STAT3-HIF-1α axis, reduces GLUT1, HK-2, and LDHA expression, and shifts macrophage metabolism from glycolytic flux toward oxidative phosphorylation. These metabolic changes well align with the phenotypic switch of macrophages from M1 to M2 type as evidenced by down-regulation of TNF-α/IL-6 and upregulation of Arg1/CD206, as well as enhancement of phagocytic capacity. Taken together, these results highlight the potential of 15KPF for resolution of inflammation and cardiac repair.

Our functional studies further validate PKM2 as the critical target of 15KPF. Mutating Cys49 to Ser49 (PKM2-C49S) or silencing PKM2 abolishes the anti-inflammatory effects of 15KPF, confirming the specificity of such interaction. Notably, the ability of 15KPF to promote mitochondrial respiration over glycolysis mirrors the effects of synthetic PKM2 activators (e.g., TEPP-46, DASA-58) 47,48. Recent therapeutic strategies targeting PKM2 have largely focused on non-covalent activators that promote its tetramerization and inhibit nuclear signaling. For instance, TEPP-46 has been shown to activate PKM2, thereby suppressing the pathogenicity of CD4⁺ T cells and ameliorating autoimmune responses 49. In another study, TEPP-46 rescued metabolic and functional outcomes following ischemia-reperfusion (I/R) injury in Mtx2-deficient mice through PKM2 activation 50. Reported PKM2 activators include derivatives such as pyrazoles, pyrrolidine-pyrazoles, phenols, benzoxazines, isoselenazolopyridiniums, phthalazines, and propiolamides. These compounds facilitate PKM2 activation, thereby reducing the availability of biosynthetic precursors and inhibiting cell proliferation and development 51. Unlike these non-covalent activators, however, 15KPF offers a unique advantage due to its covalent binding capability. It effectively blocks PKM2 nuclear translocation and subsequent transcriptional activity, while simultaneously enhancing its metabolic function. This dual mechanism—boosting metabolic activity while inhibiting inflammatory signaling—positions 15KPF as a distinctive and promising therapeutic candidate.

The translational relevance of the findings from the present study is underscored by the *in vivo* cardioprotective efficacy of 15KPF. In a murine MIRI model, 15KPF treatment reduced infarct size, improved cardiac function, and increased macrophage M2 polarization. These benefits likely stem from better phagocytic clearance of apoptotic cells and less production of pro-inflammatory cytokines, two critical processes for tissue repair 52. Importantly, 15KPF demonstrated an excellent safety profile: it exhibited no cytotoxicity in primary cardiomyocytes or bone marrow-derived macrophages (BMDMs), and no acute toxicity was observed in KM mice even at a dose 40-fold higher than the therapeutic level. Organ indices, liver function markers (AST/ALT), and histopathological evaluation of major organs revealed no abnormalities, supporting a wide therapeutic window for 15KPF (Figures S3 and S4).

Our study also expands the understanding of AA metabolism in inflammation resolution. While many AA derivatives—such as prostaglandins and leukotrienes—are traditionally considered pro-inflammatory 53, 15KPF exemplifies a growing class of anti-inflammatory and pro-resolving mediators, consistent with the emerging recognition of specialized pro-resolving lipid mediators (SPMs) 2,54. By covalently modifying PKM2, 15KPF mechanistically links AA metabolism to glycolytic reprogramming in macrophages, offering a new paradigm through which lipid-driven signaling fine-tunes immunometabolic responses. This discovery not only deepens our insight into endogenous resolution mechanisms but also opens new avenues for therapeutic intervention in inflammatory and ischemic diseases by targeting metabolic-immune crosstalk.

The limitations of this study include: While our findings establish covalent 15KPF-PKM2 interaction as a key cardioprotective mechanism, three limitations warrant attention. First, the lack of macrophage-specific PKM2 knockout or C49S mutant models precludes definitive validation the *in vivo* target specificity of 15KPF. Second, structural confirmation of the 15KPF-PKM2 conjugate via crystallography would strengthen mechanistic claims. Thirdly, a comprehensive PK study of 15KPF is needed. Future studies should address these gaps and explore 15KPF's synergy with existing therapies (e.g., anti-IL-1β agents) to optimize post-MI recovery.

## Conclusion

By elucidating covalent modulation of PKM2 by 15KPF, this work uncovers a novel metabolic-inflammatory axis in macrophage polarization. Our results position 15KPF as a promising therapeutic agent to reprogram macrophage metabolism, resolve inflammation, and enhance cardiac repair in post-MI hearts. These findings not only advance the translational potential of AA metabolites but also highlight PKM2 as a druggable node for treating inflammatory cardiovascular diseases.

## Supplementary Material

Supplementary figures and tables.

## Abbreviations

AA: Arachidonic acid
CETSA: Cellular Thermal Shift Assay
COX: cyclooxygenase
CYP450: cytochrome P450
DARTS: Drug Affinity Responsive Target Stability
DMEM: Dulbecco's Modified Eagle Medium
EETs: epoxyeicosatrienoic acids
FBS: fetal bovine serum
15KPF: 15-keto-PGF₂α
LOX: lipoxygenase
MIRI: myocardial ischemia reperfusion injury
SPMs: specialized pro-resolving mediators
PKM2: pyruvate kinase M2
PGF2α: prostaglandin F2α
15d-PGJ2: 15-deoxy-Δ12,14-prostaglandin J2
