# Supplementary material for: Crosstalk between arachidonic acid metabolism and glycolysis drives integrated metabolic-inflammatory reprogramming in macrophages
Source: Int J Biol Sci. 2026 Jan 1;22(2):771–85. doi: 10.7150/ijbs.116671 (PMC12781075; doi:10.7150/ijbs.116671)
Supplement: Supplementary file 1 — Supplementary figures and tables. [file ijbsv22p0771s1.pdf]

Crosstalk between arachidonic acid metabolism and glycolysis drives integrated metabolic-inflammatory reprogramming in macrophages

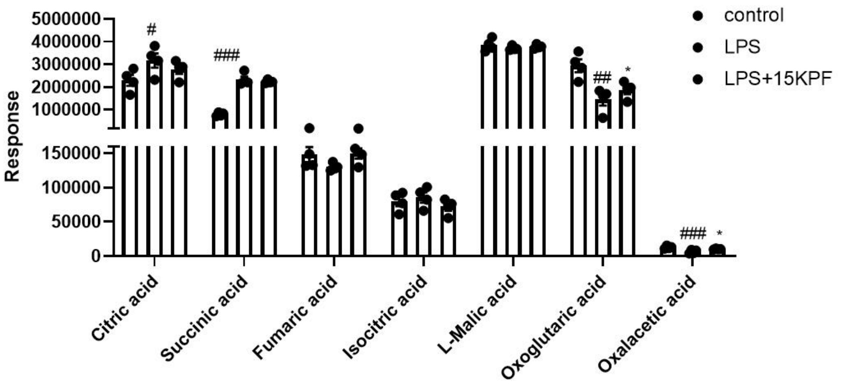

Figure S1. The effect of 15KPF on TCA metabolites. The data were expressed as means ± SD and analyzed by one-way ANOVA, followed by Dunnett's multiple comparisons test. Control vs LPS, #, $p<0.05$, ##, $p<0.01$, ###, $p<0.001$; LPS vs 15KPF treatment, *, $p<0.05$.

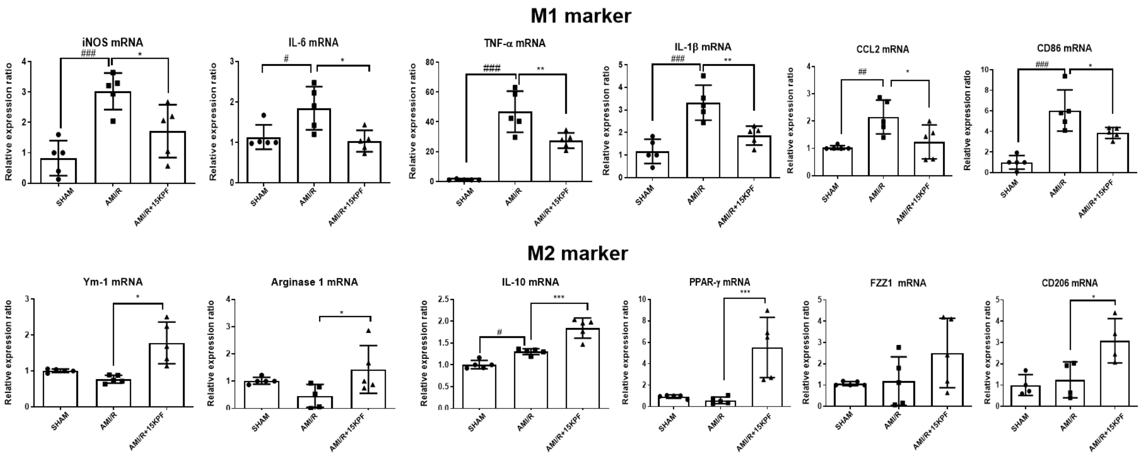

Figure S2. The effect of 15KPF on macrophage phenotypes in BMDM. BMDM were isolated from the indicated groups. The total RNA were isolated and subjected to qRT-PCR. The data were expressed as mean ± SD and analyzed one-way ANOVA, followed by Dunnett's multiple comparisons test. n = 4~5 from 3 independent experiments, Sham vs AMI/R, #, $p < 0.05$; ##, $p < 0.01$; ###, $p < 0.001$; AMI/R vs AMI/R+15KPF, *, $p < 0.05$; **, $p < 0.01$.

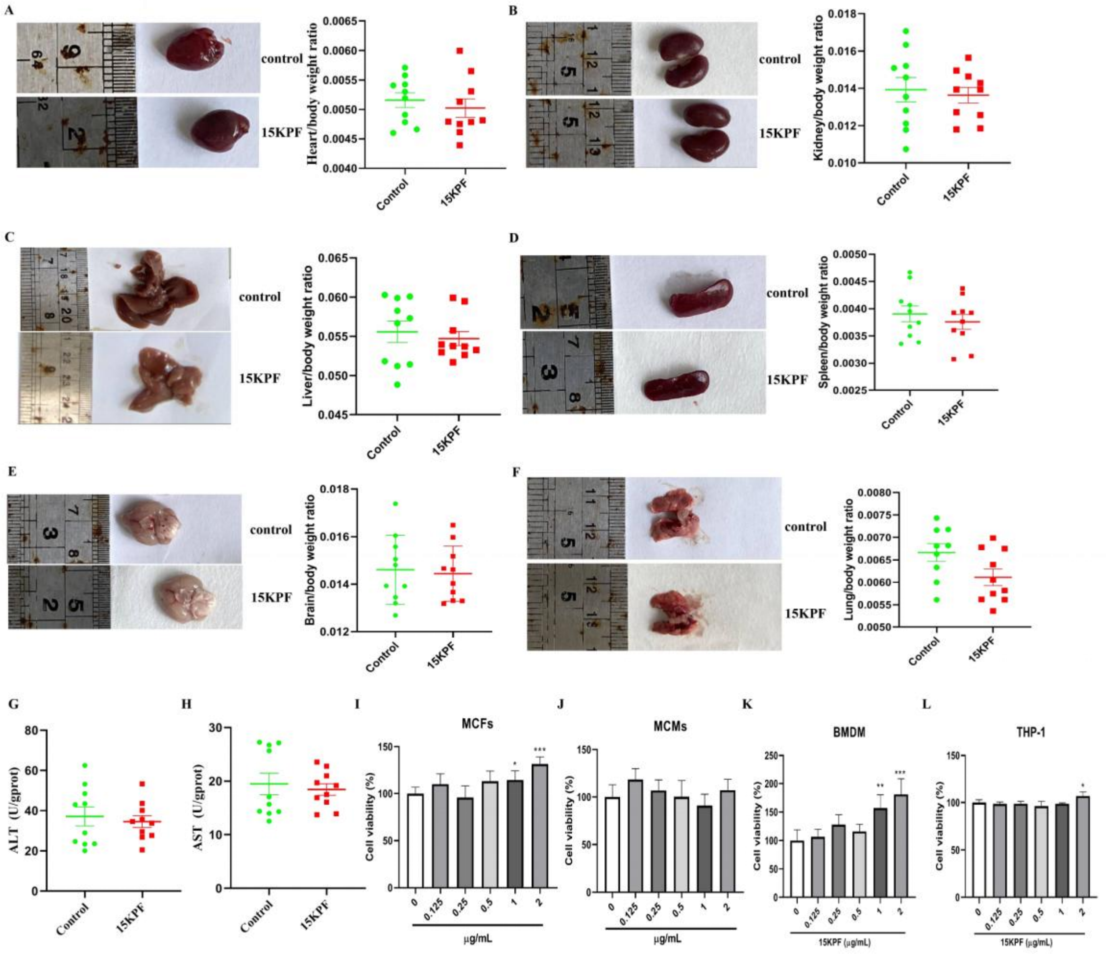

Figure S3. The toxicity of 15KPF *in vivo* and *in vitro,* related to Figure 7. (A-H)The acute toxicity test of 15KPF by measuring the organ index, ALT and AST; (I,J) The cell toxicity of 15KPF on primary cardiomyocytes and myofibroblasts. (K,L)The cell toxicity of 15KPF on primary BMDM and THP-1 cells.

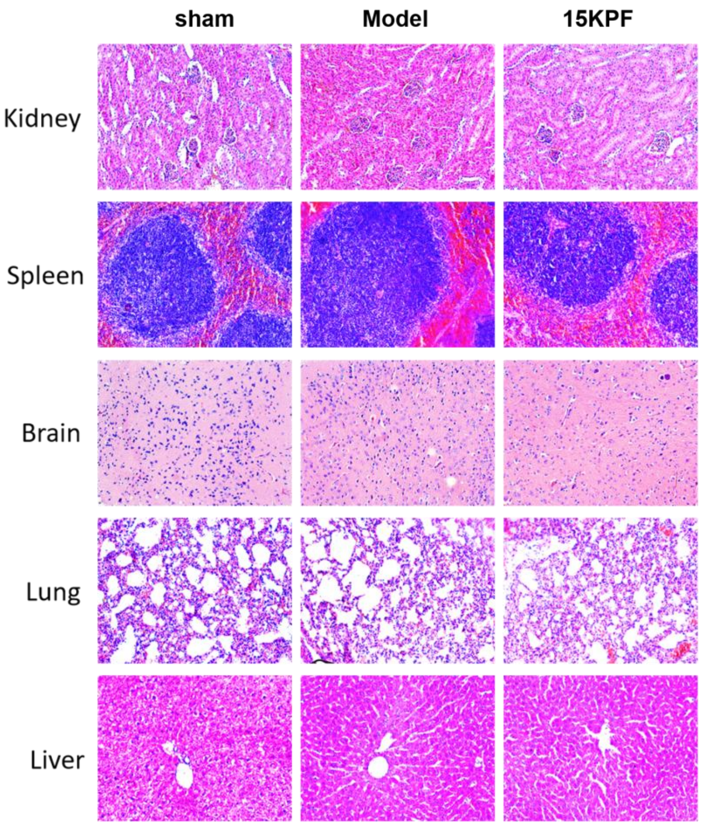

Figure S4 All histopathological sections after 15KPF administration.

KEY RESOURCES TABLE

REAGENT or RESOURCE	SOURCE	IDENTIFIER
**Antibodies**		
Cox2 (D5H5) XP® Rabbit mAb	Cell signaling Technology	Cat # 12282; RRID:AB_2571729
GAPDH (14C10) Rabbit mAb	Cell signaling Technology	Cat #5014; RRID:AB_10693448
Lamin B1 (E6M5T) Rabbit mAb	Cell signaling Technology	Cat # 17416S； RRID:AB_3095982
Phospho-Stat3 (Tyr705) (D3A7) XP® Rabbit mAb	Cell signaling Technology	Cat #52075; RRID:AB_2799407
Stat3 (79D7) Rabbit mAb	Cell signaling Technology	Cat# 4904S; RRID:AB_331269
PKM2 (D78A4) XP® Rabbit mAb	Cell signaling Technology	Cat# 4053S; RRID:AB_1904096
PKM2-specific Rabbit mAb	ABclonal	Cat# A20991; RRID:AB_3095983
Phospho-PKM2 (Tyr105) Antibody #3827	Cell signaling Technology	Cat# 3827S; RRID:AB_1950369
Anti-GLUT1 (rabbit monoclonal)	Cell Signaling Technology	Cat# 12939S; RRID:AB_2687899
Anti-HK2 (rabbit polyclonal)	Abclonal	Cat# A0994; RRID:AB_2757513
Anti-LDHA (rabbit polyclonal)	Cell Signaling Technology	Cat# 2012S; RRID:AB 3095984
Anti- β -actin (rabbit polyclonal)	Thermo Fisher Scientific	Cat# PA1-183; RRID:AB_2539914
Mouse monoclonal anti-HIF1 α	Santa Cruz	Cat# sc-13515; RRID:AB_627723
Mouse monoclonal anti-Desmin	Santa Cruz	Cat# sc-65983; RRID:AB_1122192
Mouse monoclonal anti-Arginase 1	Santa Cruz	Cat# sc-166920; RRID:AB_10609486
Mouse monoclonal anti-CD206	Santa Cruz	Cat# sc-376108; RRID:AB_10987732
Rabbit monoclonal	Abcam	Cat# ab283654; RRID:AB_2922954

anti-CD68		
Rabbit monoclonal anti-iNOS	Abcam	Cat# ab178945; RRID:AB_2861417
Alexa Fluor 594-goat anti-rabbit IgG secondary antibody	Thermo Fisher Scientific	Cat# A-11012; RRID:AB_2534079
Alexa Fluor 488-goat anti-mouse IgG secondary antibody	Thermo Fisher Scientific	Cat# A-11001; RRID:AB_2534069
Goat anti-mouse IgG-HRP	Santa Cruz	Cat# sc-2005; RRID:AB_631736
Anti-Rabbit IgG (whole molecule)	Sigma-Aldrich	Cat# A0545; RRID:AB_257896
**Chemicals and Recombinant protein**		
ω-Alkynyl arachidonic acid	Cayman Chemical	Cat# 10538; CAS: 1219038-32-0
15-keto-PGF2α	Cayman Chemical	Cat# 10007227; CAS: 35850-13-6
Recombinant human PKM2 protein	Abcam	Cat# ab89364
Lipopolysaccharides from Escherichia coli O111:B4	Sigma-Aldrich	Cat# L4391
DAPI	Thermo Fisher Scientific	Cat# D1306
Hematoxylin Solution	Sigma-Aldrich	Cat# HHS32-1L
Eosin Y-solution 0.5% alcoholic	Sigma-Aldrich	Cat# 102439
Azide-PEG3-biotin conjugate	Sigma Aldrich	Cat# 762024; CAS: 875770-34-6
**Critical Commerical Assays**		
Pyruvate kinase activity assay kit	Biovision	Cat# K709-100
NE-PERTM Nuclear and Cytoplasmic extraction reagents	Life technologies	Cat# 78835
Seahorse XF Glycolysis Stress Test Kit	Agilent Technologies	Cat# 103020-100
Seahorse XF Cell Mito Stress Test Kit	Agilent Technologies	Cat# 103015-100

Amersham™ ECL™ Select Western Blotting Detection Reagent	GE Healthcare	Cat# RPN2235
TRIzol™ Reagent	Thermo Fisher Scientific	Cat # 15596026
RevertAid first-strand cDNA synthesis kit	Thermo Fisher Scientific	Cat# K1622
SYBR Green mix	QIAGEN	Cat# 204056
**Recombinant DNA**		
pLHCX-Flag-mPKM2	Addgene	Cat# 42512; RRID: Addgene_42512
**Oligonucleotides**		
Mm_Il6_1_SG	QIAGEN	Cat# QT00098875
Mm_Il1b_2_SG	QIAGEN	Cat# QT01048355
Mm_Nos2_1_SG	QIAGEN	Cat# QT00100275
Mm_Tnf_1_SG	QIAGEN	Cat# QT00104006
Mm_Ccl2_1_SG	QIAGEN	Cat# QT00167832
Mm_Cxcl10_1_SG	QIAGEN	Cat# QT00093436
Mm_Arg1_1_SG	QIAGEN	Cat# QT00134288
Mm_Il10ra_1_SG	QIAGEN	Cat# QT00112742
**Experimental Models: Cell lines**		
Murine macrophage cell line RAW264.7	ATCC	Cat# TIB-71; RRID:CVCL_0493
H9c2 cells	ATCC	Cat# CRL-1446; RRID:CVCL_0286
**Experimental models: Organisms/strains**		
C57BL/6J mice	Jackson Laboratory animal	RRID:IMSR_JAX:000664
**Software and Algorithms**		
Image J	National Institutes of Health	https://imagej.nih.gov/ij/; RRID:SCR_003070
Prism 5	GraphPad	https://www.graphpad.com/scientificsoftware/prism/; RRID:SCR_002798

Table S1 Multiple-reaction monitoring transition ions for AA metabolites of interest

Analyte	Parent ions (mass to-charge ratio) negative	Fragmentation ions (mass-to-charge ratio)
Alkynyl-PGE2	347	267
PGE2	351	271
Alkynyl-PGF2α	349	193
PGF2α	353	193
Alkynyl-PGE3	345	265
PGE3	349	269
Alkynyl-LTB4	331	195
LTB4	335	195
Alkynyl-15-HETE	315	253
15-HETE	319	257
Alkynyl-11-HETE	315	167
11-HETE	319	167
Alkynyl-5-HETE	315	115
5-HETE	319	115
Alkynyl-15-keto-PGF2α	347	213
15-keto-PGF2α	351	213
Alkynyl-13,14-dihydro-15-oxo-PGF2α	349	211
13,14-dihydro-15-oxo-PGF2α	353	211
Alkynyl-TXB2	365	169
TXB2	369	169
Alkynyl-LTB4	331	191
LTB4	335	195
